# Creation of a machine learning-based prognostic prediction model for various subtypes of laryngeal cancer

**DOI:** 10.1038/s41598-024-56687-x

**Published:** 2024-03-18

**Authors:** Wei Wang, Wenhui Wang, Dongdong Zhang, Peiji Zeng, Yue Wang, Min Lei, Yongjun Hong, Chengfu Cai

**Affiliations:** 1 Department of Otolaryngology-Head and Neck Surgery, Zhongshan Hospital of Xiamen University, School of Medicine, Xiamen University, Xiamen, China; 2 School of Medicine, Xiamen University, Xiamen, China; 3 Otorhinolaryngology Head and Neck Surgery, Xiamen Medical College Affiliated Haicang Hospital, Xiamen, China

**Keywords:** Laryngeal carcinoma, Survival analysis, Machine learning algorithm, Individual prediction, Cancer, Metastasis

## Abstract

Depending on the source of the blastophore, there are various subtypes of laryngeal cancer, each with a unique metastatic risk and prognosis. The forecasting of their prognosis is a pressing issue that needs to be resolved. This study comprised 5953 patients with glottic carcinoma and 4465 individuals with non-glottic type (supraglottic and subglottic). Five clinicopathological characteristics of glottic and non-glottic carcinoma were screened using univariate and multivariate regression for CoxPH (Cox proportional hazards); for other models, 10 (glottic) and 11 (non-glottic) clinicopathological characteristics were selected using least absolute shrinkage and selection operator (LASSO) regression analysis, respectively; the corresponding survival models were established; and the best model was evaluated. We discovered that RSF (Random survival forest) was a superior model for both glottic and non-glottic carcinoma, with a projected concordance index (C-index) of 0.687 for glottic and 0.657 for non-glottic, respectively. The integrated Brier score (IBS) of their 1-year, 3-year, and 5-year time points is, respectively, 0.116, 0.182, 0.195 (glottic), and 0.130, 0.215, 0.220 (non-glottic), demonstrating the model's effective correction. We represented significant variables in a Shapley Additive Explanations (SHAP) plot. The two models are then combined to predict the prognosis for two distinct individuals, which has some effectiveness in predicting prognosis. For our investigation, we established separate models for glottic carcinoma and non-glottic carcinoma that were most effective at predicting survival. RSF is used to evaluate both glottic and non-glottic cancer, and it has a considerable impact on patient prognosis and risk factor prediction.

## Introduction

Laryngeal carcinoma, which makes up 20% of all malignant tumors of the head and neck, is a prevalent type of these malignancies^1,2^. Laryngeal cancer is estimated to affect over 1,700,000 people annually, and 123,000 people died from it in 2019—86% of whom were men^3,4^. The majority of pathological kinds of laryngeal cancer, which include squamous cell carcinoma, are believed to be associated with smoking, alcohol use, human papillomavirus infection, and air pollution. Early detection and treatment are especially crucial for laryngeal cancer because it has an excellent prognosis and quality of life. Advanced laryngeal cancer can be treated with definitive Radiation therapy, combination chemotherapy, or adjuvant radiotherapy and total laryngectomy in addition to surgery, radiotherapy, chemotherapy, and other comprehensive treatments^5,6^. Different stages, forms, and treatments of laryngeal cancer have varying chances of survival, and the overall patient's 5-year relative survival rate is 64%^2^. Clinically, we use the conventional TNM staging and the 5-year survival rate to develop an overall understanding of the prognosis of patients, but it is challenging to quantify and depict it^7^. The conventional Cox proportional hazards (CoxPH) model is a prominent prediction model used to accomplish this goal, but it has limitations since it is based on the presumption that there is a linear relationship between survival outcomes and clinical variables^8,9^. At the same time, the standard survival analysis is unable to forecast an individual's survival prognosis with any degree of precision. The creation and prediction of tumor-related survival models now uses a large number of sophisticated Machine learning techniques (MLTs). When it comes to predicting the prognosis of tumor patients, MLTs—which include Random Survival Forest (RSF), Gradient Boosting Machine (GBM), eXtreme Gradient Boosting (XGBoost), and deep learning model Deepsurv—have been demonstrated to be more accurate than CoxPH model^10–14^. Studies on the equivalent machine learning of laryngeal cancer to create a survival model have been conducted concurrently^15^. Unfortunately, there are two flaws in the way that current research predicts the prognosis and survival of laryngeal cancer. First off, the performance of these models is constrained by the small sample size and homogeneity of the prediction algorithms. Furthermore, the lymph node metastatic rates of supraglottic and subglottic laryngeal carcinoma (non-glottic laryngeal carcinoma), which were 19.9% and 8.0% respectively, were higher. This contributed to the varied survival rates for glottic and non-glottic laryngeal cancer^16,17^. How to accurately predict the survival of patients with different types of laryngeal cancer has become a key problem. Therefore, in this study, we selected different types of laryngeal cancer patients using SEER database data, and developed survival models for glottic and non-glottic cancer, describing the main factors, to predict the survival of patients with laryngeal cancer more accurately. We develop the survival model using data from the SEER database, and compare the CoxPH model with four widely used machine learning techniques. Last but not least, we apply the model to forecast each person's prognosis, which aligns more with clinical application.

## Methods

### Data collection

The Surveillance, Epidemiology, and End Results Program (SEER) database was used to gather the study's data (Incidence-Seer Research Plus Data 17 Registries Nov 2021 Sub). Using the SEER*Stat program (version 8.4.2), we retrieved individuals who had been given a larynx carcinoma diagnosis by the third edition of the International Classification of Oncology Diseases (ICD-O-3). The period frame covers instances handled between 2000 and 2019. The following were the inclusion requirements: The behavior was identified as malignant and encoded by position and shape as "larynx".

### Data clarity

In total, 54,613 patients with primary laryngeal malignant tumors were included. The median follow-up duration of the sample in this study is 38 months. We used the following exclusion criteria to clean up the data: (1) Patients with limited follow-up information; (2) Patients without T stage (AJCC7), N stage (AJCC7), M stage (AJCC7), or AJCC stage grade information.

### Feature selection

We selected variables that were directly related to the clinic, such as age, race, and gender, based on clinical experience. We chose the T stage, N stage, M stage, AJCC stage (AJCC stage 7), tumor size, and pathological categorization to assess the patient's health. Finally, to evaluate the patient's treatment plans, we also included radiation therapy, surgery, and chemotherapy.

### Models for survival analysis

A classic model for survival analysis, the Cox proportional hazards (CoxPH) model has been the most commonly applied multifactor analysis technique in survival analysis to date^18,19^.

CoxPH is a statistical technique for survival analysis, which is mainly used to study the relationship between survival time and one or more predictors. The core of the model is the proportional risk hypothesis.

It is expressed as *h(t|x)* = *h0 (t) exp (β|x)*, h(t|x) is the instantaneous risk function under the given covariable x, h0 (t) is the baseline risk function, on the other hand, exp (β x) represents the multiplicative effect of covariates on risk.

The random survival forest (RSF) model is an extremely efficient integrated learning model that can handle complex data linkages and is made up of numerous decision trees^20^.

RSF can improve the accuracy and robustness of the prediction, but it does not have a single expression because it is an integrated model consisting of multiple decision trees^21^. RSF constructs 1000 trees and calculates the importance of variables. To find the optimal model parameters, we adjust three key parameters: the maximum number of features of the tree (mtry), the minimum sample size of each node (nodesize), and the maximum depth of the tree (nodedepth). The values of these parameters are set to mtry from 1 to 10, nodesize from 3 to 30, and nodedepth from 3 to 6. We use a random search strategy (RandomSearch) to optimize the parameters. To evaluate the performance of the model under different parameter configurations, we use tenfold cross-validation and use C-index (ConcordanceIndex) as the evaluation index. The purpose of this process is to find the parameter configuration that can maximize the prediction accuracy of the model through many iterations.

One of the integrated learning methods called Boosting is the gradient boosting machine (GBM) model, which constructs a strong prediction model by combining several weak prediction models (usually decision trees). At each step, GBM adds a new weak learner by minimizing the loss function. The newly added model is trained to reduce the residual generated in the previous step, and the direction is determined by the gradient descent method. It can be expressed as *F*_*m*+*1*_*(x)* = *F*_*m*_*(x)* + *α*_*m*_*h*_*m*_*(x)*. Where the F_m_(x) is a weak model newly added, and the α_m_ is the learning rate.

XGBoost is an efficient implementation of GBM, especially in optimizing computing speed and efficiency. To reuse the learner with the highest performance, it linearly combines the base learner with various weights^22^. eXtreme Gradient Boosting (XGBoost) is an optimization of the Gradient Boosting Decision Tree (GBDT), which boosts the algorithm's speed and effectiveness^23^. The neural network-based multi-task logic regression model developed by Deepsurv outperforms the conventional linear survival model in terms of performance^24^. DeepSurv uses a deep neural network to simulate the Cox proportional hazard model. Therefore, deepsurv can be expressed as *h(t|x)* = *h*_*0*_* (t) exp (g(x))*, Where the g (x) is the output of the neural network, which represents the linear combination of the covariable x^8^.

### Model training and validation

We categorize five models to adapt to various variable screening techniques used with various models. The RSF, GBM, and XGBoost models are screened using the least absolute shrinkage and selection operator (LASSO) regression analysis, while the CoxPH model is screened using the traditional Univariate and multivariate Cox regression analysis^25–27^.

In contrast, the Deepsurv model can automatically extract features and handle high-dimensional data and nonlinear relationships, so variable screening is not necessary^28^. We randomly split the data set into t and v datasets (training set and validation set) and test set in the ratio of 9:1 using spss (version 26) to further illustrate the model's dependability. Randomly selected 10% of the data as external verification. Once more, the ratio of 7:3 is used to divide the training set and validation set, and for both splits, the log-rank test is used to evaluate any differences between the two cohorts. The mlr3 package of R (version 4.2.2) uses the grid search approach to fine-tune the hyperparameters in the RSF, GBM, and XGBoost models in the validation set and chooses the most beneficial hyperparameters to build the survival model once the variables have been filtered following the aforementioned stages. Finally, the Deepsurv model is constructed using the Python (version 3.9) sksurv package, and the model is additionally optimized using grid search.

### Model evaluation and interpretation

We used the integrated Brier score (IBS), which is appropriate for 1-year, 3-year, and 5-year time points, as the major assessment metric when evaluating the prediction performance of the model in the test set. In addition, the calibration curve is drawn and the conventional time-dependent receiver operating characteristic (ROC) curve as well as the area under the curve (AUC) (1 year, 3 years, and 5 years) are compared. By calculating the clinical net benefit to address the actual needs of clinical decisions, Decision Curve Analysis (DCA), a clinical evaluation prediction model, incorporates the preferences of patients or decision-makers into the analysis. Calculating the various clinicopathological characteristics is also required for the prognosis of contribution. We visualized the survival contribution of several clinicopathological characteristics for 1-year, 3-years, and 5-years using The Shapley Additive Explanations (SHAP) plot.

### The particular prediction

Clinically speaking, various individuals require personalized care. Consequently, it is crucial to estimate the likelihood that a single patient will survive. The survival probability of a certain patient is predicted using the ggh4x package of R (version 4.2.2), along with the contribution of several clinicopathological characteristics to survival. This has major clinical work implications.

## Results

### Baseline characteristics

**Figure 1:**
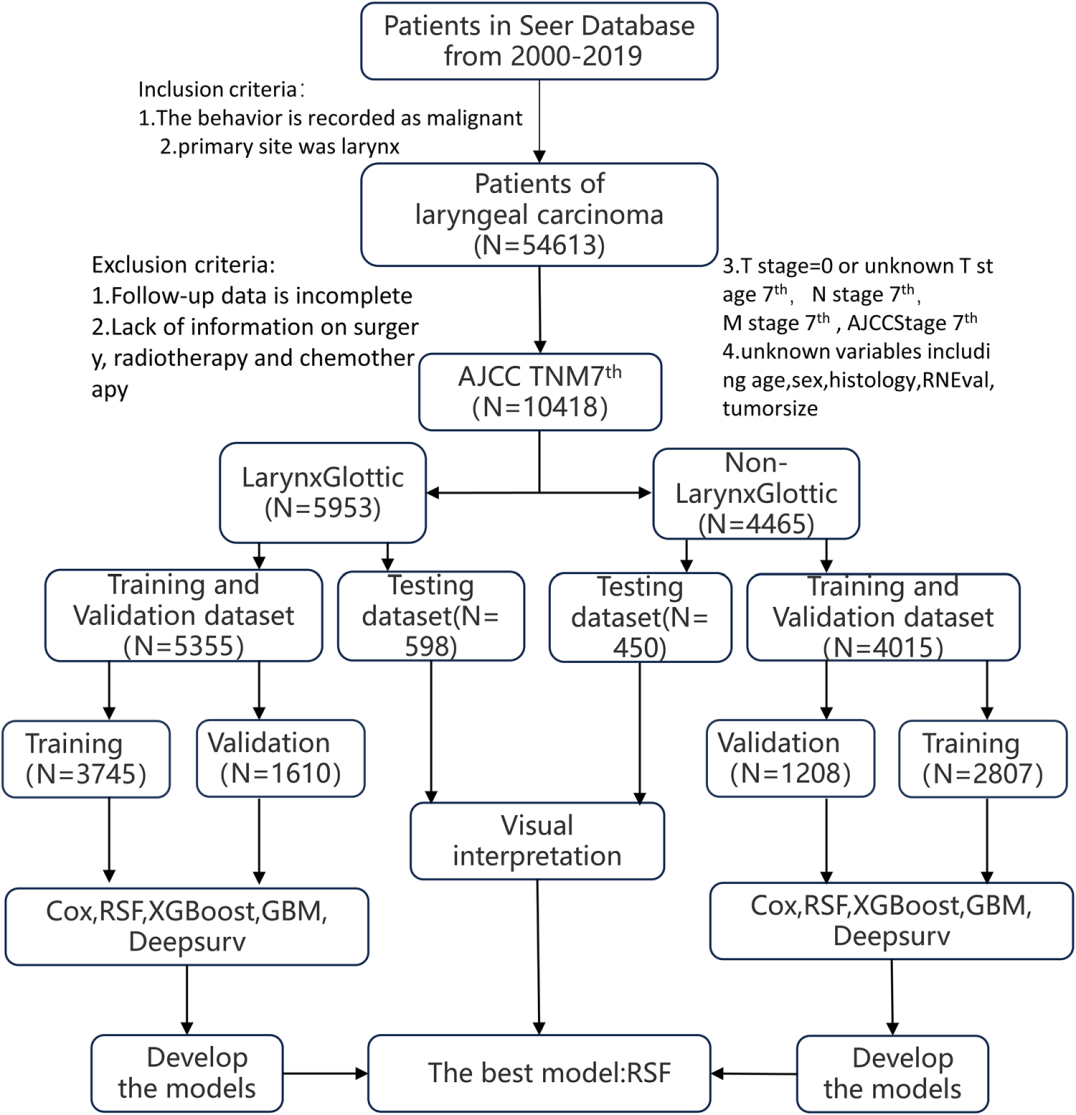
Diagrammatic sketch of study design.

**Figure 2:**
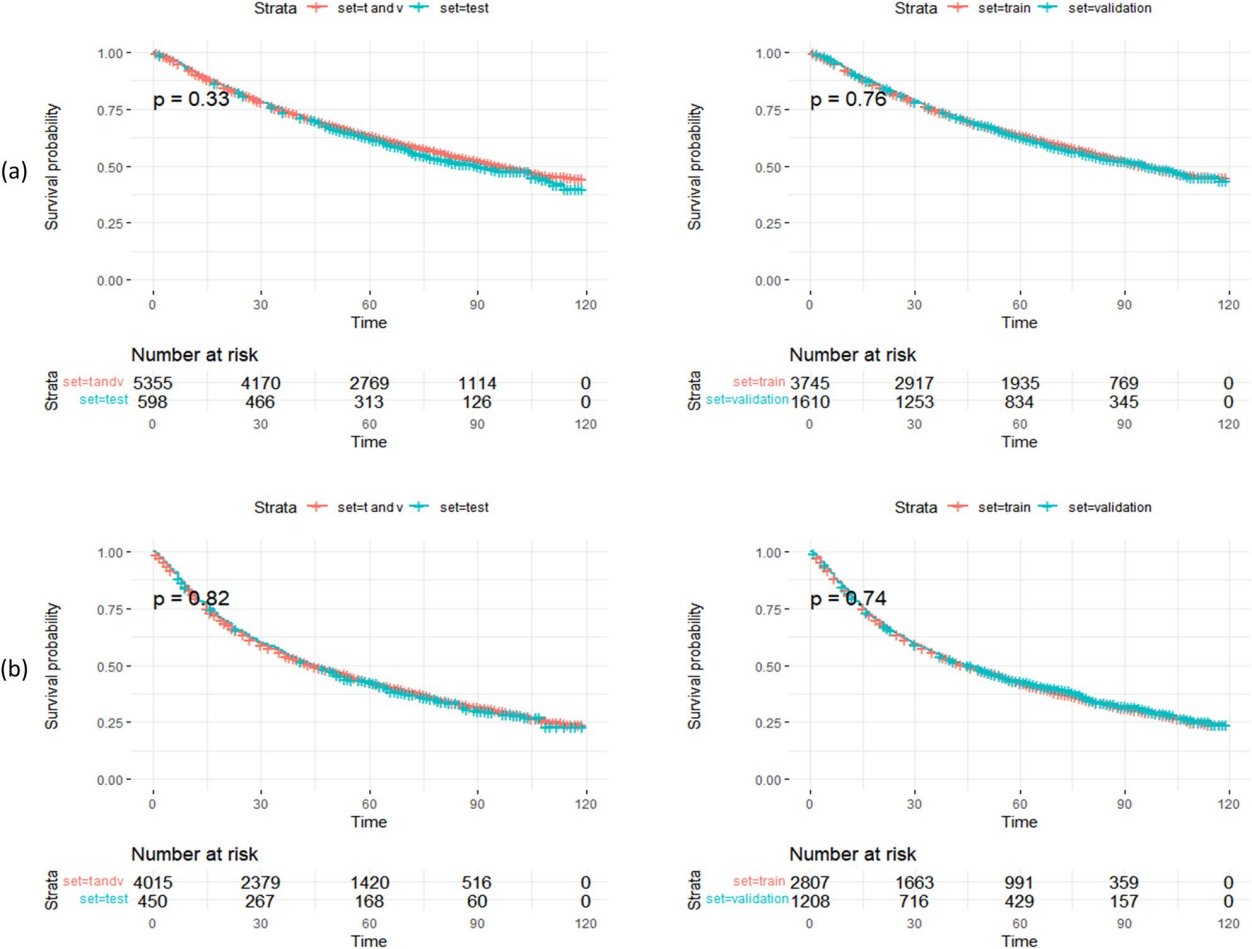
The t(train), v(validation), and test cohorts' Kaplan–Meier curves. The log-rank test revealed no statistically significant difference between the two cohorts' survival rates (P > 0.05). Unit of time: month. (**a**) glottic carcinoma, (**b**) non-glottic carcinoma.

The information of 54,613 patients was included. After data cleaning, there were 5953 patients with glottic carcinoma and 4465 patients with non-glottic (supraglottic and subglottic) cancer as a result of the aforementioned exclusion criteria. Figure 1 shows specific cleaning procedures. Table 1 displays the clinical and clinicopathological characteristics of these patients as well as the relevant categorization ratio. In Fig. 2, the survival curve was displayed after patients with glottic and non-glottic cancer were divided into training and validation datasets and testing datasets, respectively.

**Table 1:** The information for laryngeal carcinoma patients in the training set and the validation set.

Glottic laryngeal carcinoma						
Variable	Training cohort (n,%) N = 3745	Validation cohort (n, %) N = 1610	P value	Title 1	Title 2	Title 3
Age (year)			0.954	Entry 1	Data	Data
15–49	238 (6.3)	96 (6.1)				
50–59	774 (20.4)	323 (20.6)				
60–69	1189 (31.4)	502 (32.1)				
70–79	1014 (26.8)	420 (26.8)				
> 79	574 (15.1)	225 (14.4)				
Sex			0.066			
Female	495 (13.1)	176 (11.2)				
Male	3294 (86.9)	1390 (88.8)				
Histology			0.383			
Other	95 (2.5)	33 (2.1)				
Squamous cell carcinoma	3694 (97.5)	1533 (97.9)				
Tumor size(cm)			0.559			
< 1	1148 (30.3)	472 (30.1)				
< 2	11 (0.3)	2 (0.1)				
> 2	2 (0.1)	0 (0)				
Other	2628 (69.4)	1092 (69.7)				
RNEval			0.987			
No examination	3452 (91.1)	1433 (91.5)				
Endoscopic examination	73 (1.9)	30 (1.9)				
Autopsy	1 (0)	0 (0)				
RLN removed	225 (5.9)	86 (5.5)				
Endoscopic examination	17 (0.4)	7 (0.4)				
Removed. pathologic	17 (0.4)	8 (0.5)				
Other	4 (0.1)	2 (0.1)				
AJCC T			0.647			
T1	2123 (56.0)	878 (56.1)				
T2	823 (21.7)	357 (22.8)				
T3	504 (13.3)	205 (13.1)				
T4	339 (8.9)	126 (8.0)				
AJCC N			0.345			
N0	3416 (90.2)	1423 (90.9)				
N1	157 (4.1)	71 (4.5)				
N2a	13 (0.3)	5 (0.3)				
N2b	112 (3.0)	29 (1.9)				
N2c	81 (2.1)	34 (2.2)				
N3	10 (0.3)	4 (0.3)				
AJCC M			0.712			
M0	3751 (99.0)	1552 (99.1)				
M1	38 (1.0)	14 (0.9)				
AJCC stage			0.8			
Stage I	2072 (54.7)	867 (55.4)				
Stage II	748 (19.7)	323 (20.6)				
Stage III	504 (13.3)	204 (13.0)				
Stage IVa	413 (10.9)	154 (9.8)				
Stage IVb	14 (0.4)	4 (0.3)				
Stage IVc	38 (1.0)	14 (0.9)				
Surgery			0.187			
No	2457 (64.8)	1045 (66.7)				
Yes	1332 (35.2)	521 (33.3)				
Radiotherapy			0.755			
No	100 (2.6)	39 (2.5)				
Yes	3746 (97.4)	1521 (97.5)				
Chemotherapy			0.515			
No	3014 (79.5)	1258 (80.3)				
Yes	775 (20.5)	308 (19.7)				

Non-glottic laryngeal carcinoma						
Variable	Training cohort (n, %) N = 2807	Validation cohort (n, %) N = 1208	P value	Title 1	Title 2	Title 3
Age (year)			0.144	Entry 1	Data	Data
15–49	232 (8.1)	71 (6.1)				
50–59	885 (31.0)	390 (33.7)				
60–69	1004 (35.1)	406 (35.1)				
70–79	561 (19.6)	225 (19.5)				
> 79	177 (6.2)	64 (5.5)				
Sex			0.611			
Female	840 (29.4)	349 (30.2)				
Male	2019 (70.6)	807 (69.8)				
Histology			0.097			
Other	101 (3.5)	29 (2.5)				
Squamous cell carcinoma	2758 (96.5)	1127 (97.5)				
Tumor size(cm)			0.597			
< 1	1676 (58.6)	701 (60.6)				
< 2	11 (0.4)	5 (0.4)				
> 2	7 (0.2)	4 (0.3)				
Other	1165 (40.7)	446 (38.6)				
RN Eval			0.137			
No examination	2196 (76.8)	888 (76.8)				
Endoscopic examination	243 (8.5)	115 (9.9)				
Autopsy	2 (0.1)	0 (0)				
RLN removed	364 (12.7)	130 (11.2)				
Endoscopic examination	31 (1.1)	7 (0.6)				
Removed. pathologic	18 (0.6)	14 (1.2)				
Other	5 (0.2)	2 (0.2)				
AJCC T			0.78			
T1	454 (15.9)	188 (16.3)				
T2	953 (33.3)	395 (34.2)				
T3	994 (34.8)	382 (33.0)				
T4	458 (16.0)	191 (16.5)				
AJCC N			0.431			
N0	1341 (46.9)	514 (44.5)				
N1	461 (16.1)	215 (18.6)				
N2a	61 (2.1)	27 (2.3)				
N2b	387 (13.5)	162 (14.0)				
N2c	531 (18.6)	211 (18.3)				
N3	78 (2.7)	27 (2.3)				
AJCC M			0.825			
M0	2756 (96.4)	1116 (96.5)				
M1	103 (3.6)	40 (3.5)				
AJCC stage			0.886			
Stage I	297 (10.4)	115 (9.9)				
Stage II	479 (16.8)	188 (16.3)				
Stage III	763 (26.7)	330 (28.5)				
Stage IVa	1132 (39.6)	446 (38.6)				
Stage IVb	85 (3.0)	37 (3.2)				
Stage IVc	103 (3.6)	40 (3.5)				
Surgery			0.162			
No	2159 (75.5)	897 (77.6)				
Yes	700 (24.5)	259 (22.4)				
Radiotherapy			0.895			
No	121 (4.2)	50 (4.3)				
Yes	2738 (95.8)	1106 (95.7)				
Chemotherapy			0.576			
No	1044 (36.5)	433 (37.5)				
Yes	1815 (63.5)	723 (62.5)				

*RN Eval* regular nodes evaluation, *AJCC* American Joint Committee on Cancer.

### Feature selection and model construction

**Figure 3:**
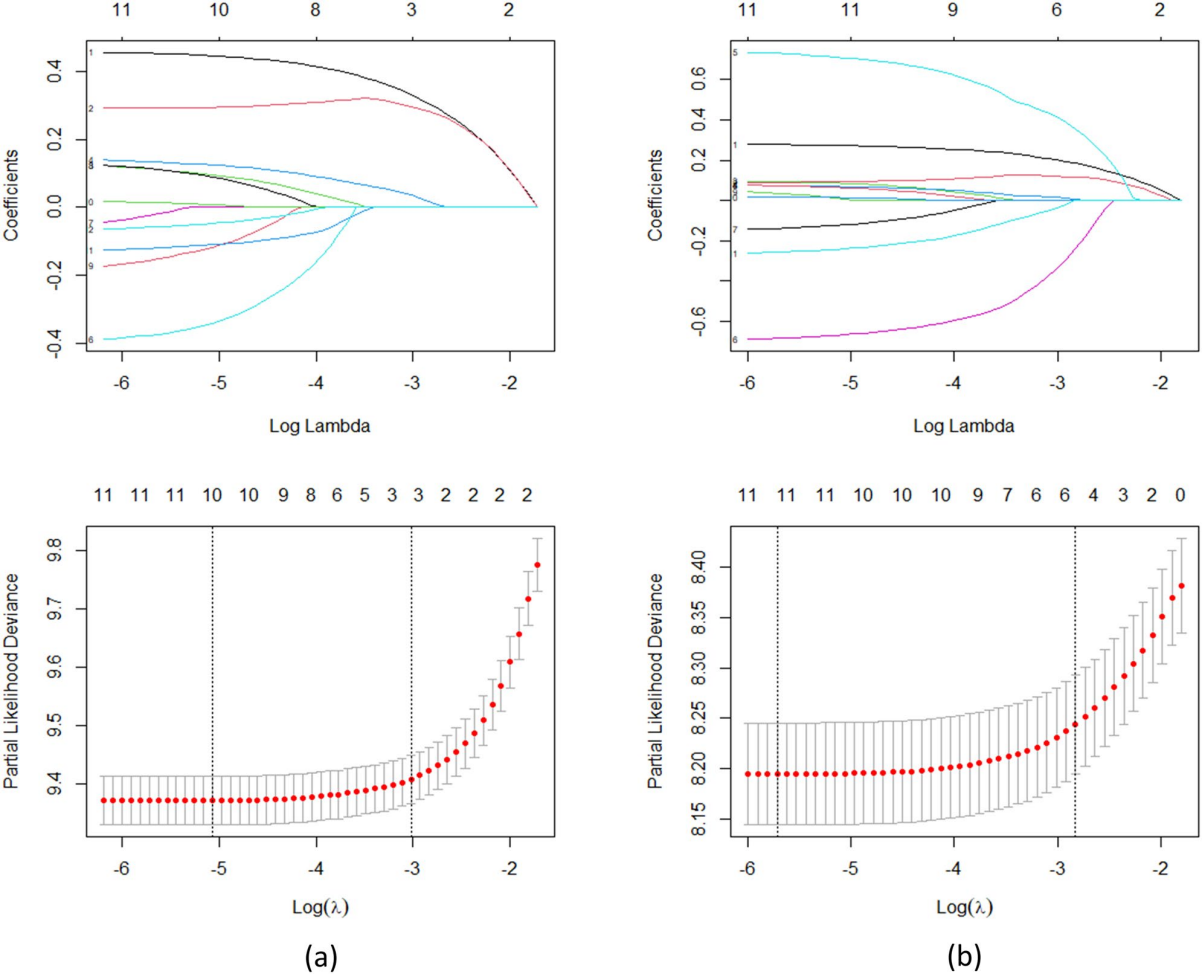
The clinicopathological characteristics of the machine learning model were examined using the least absolute shrinkage and selection operator (LASSO) regression. (**a**) glottic carcinoma, (**b**) non-glottic carcinoma.

Age, histology, tumor size, RN Eval (Regular Nodes Evaluation), AJCC T, AJCC N, AJCC M, AJCC Stage, surgery, and chemotherapy were the 10 significant mutations identified in the univariate Cox regression analysis for Glottic Carcinoma. Following multivariate Cox regression, age, AJCC T, AJCC N, AJCC Stage, and surgery were the final 5 effective variables to be included. Similarly, the effective variables of univariate Cox regression analysis and multivariate Cox regression analysis of non-glottic laryngeal carcinoma were age, sex, tumor size, RN Eval, AJCC T, AJCC N, AJCC M, AJCC Stage, surgery, radiotherapy and age, sex, AJCC Stage, surgery, radiotherapy. Table S1 displays the outcomes of the univariate and multivariate Cox regression. Machine learning characteristic variables (Fig. 3) were chosen using lasso regression analysis based on the lowest standard. A total of 10 efficient variables were chosen for glottic carcinoma: age, sex, histology, AJCC T, AJCC N, AJCC Stage, RN Eval, radiotherapy, surgery, and tumor size. The 11 effective variables were chosen for non-glottic carcinoma: age, sex, histology, AJCC T, AJCC N, AJCC M, AJCC Stage, chemotherapy, radiotherapy, surgery, and tumor size.

### Constructing and evaluating survival analysis models

**Figure 4:**
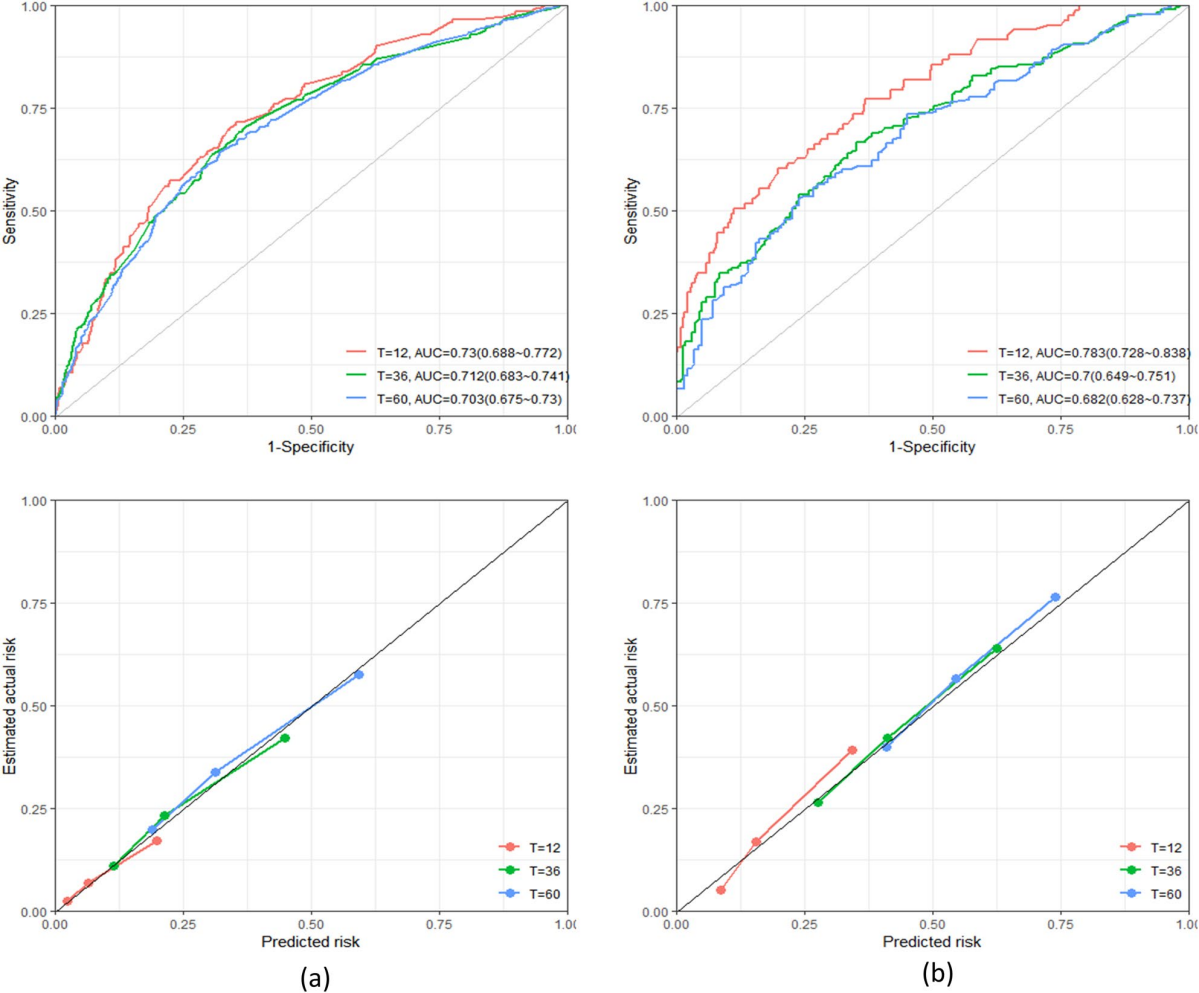
The chart "RSF Model for predicting 3-and 5-year Survival rates of Laryngeal Cancer patients: calibration Curve and time-dependent ROC Curve" shows the calibration curve and time-dependent ROC curve of RSF model for predicting 3-and 5-year survival rates of laryngeal cancer patients. The calibration curve shows the consistency between the predicted survival rate and the actual survival rate, while the ROC curve provides the performance of the model under different discriminant thresholds. Month is the unit of time. (**a**) glottic carcinoma, (**b**) non-glottic carcinoma.

**Figure 5:**
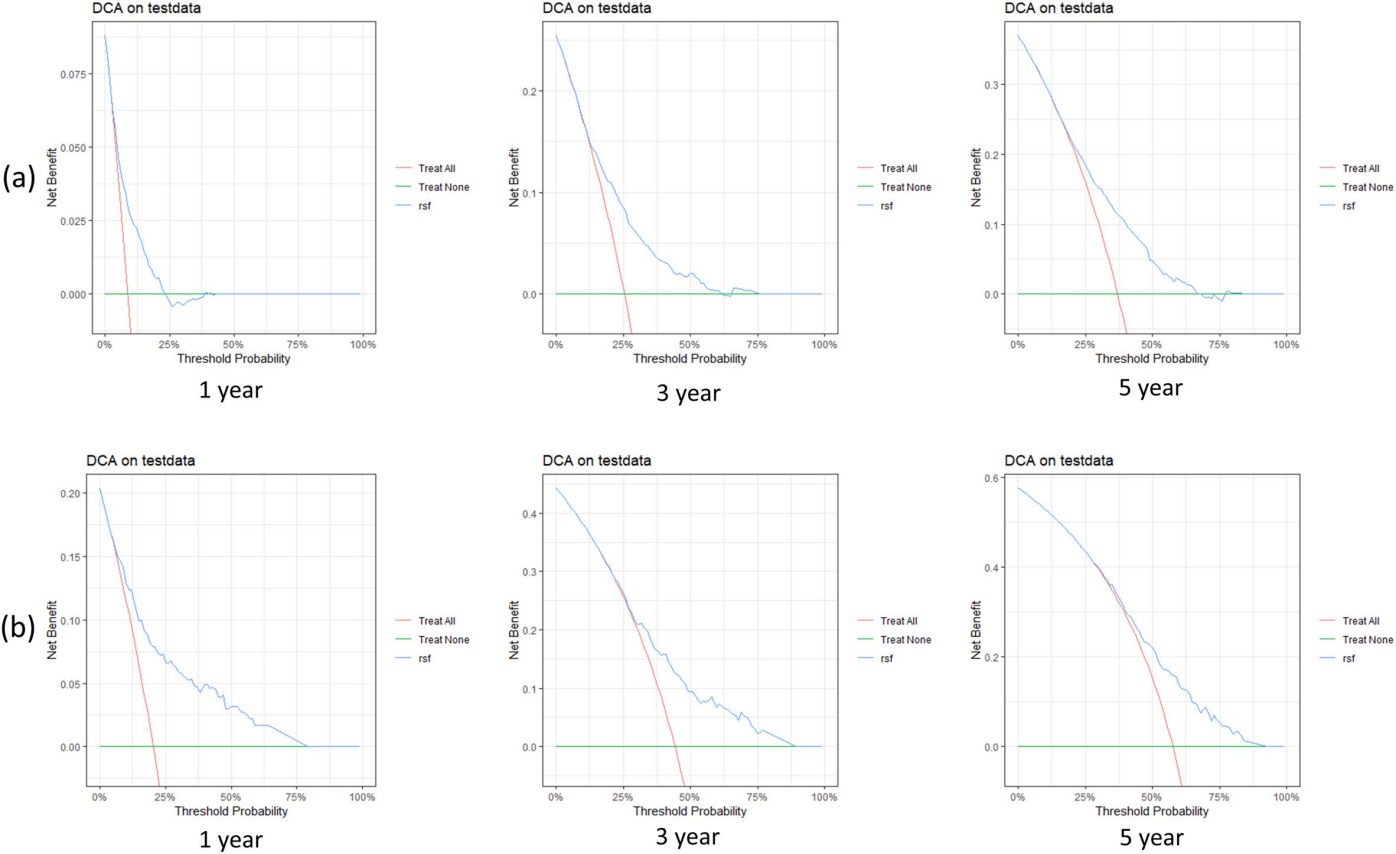
The 3-year and 5-year decision-making curves are based on the RSF model. The decision curve shows the net benefit of the model prediction under different patient risk thresholds. By comparing the net income of model prediction with and without model prediction under a specific risk threshold, the application value of the model in clinical decision-making can be evaluated. In the figure, the horizontal axis represents the decision threshold and the vertical axis represents the net income. The points on the curve represent the relative net income that the model can predict under a given risk threshold. Ideally, the higher the curve, the greater the net income provided by the model within a wider threshold range, that is, the higher the value of the model in clinical application. (**a**) glottic carcinoma, (**b**) non-glottic carcinoma.

We built the CoxPH model, RSF model, GBM model, and XGBoost model for glottic and non-glottic cancer by the outcomes of multivariate Cox regression analysis and lasso regression analysis, respectively. The Deepsurv model does not require variable screening, hence all 12 variables are used in the model during model development. All survival models are trained to roughly estimate their performance and stability range using Ten-fold cross-validation C-index and IBS. Following the visual examination of the test set, we eventually obtained the following secondary outcomes: 1-year, 3-year, and 5-year ROC curve, calibration curve, C-index, and 1-year, 3-year, and 5-year IBS (Table 2 and Fig. 4). The indicators of the training set are shown in Table S2. All models have IBS that are less than 0.25, which suggests that they can be calibrated well. Figure 5 displays the 1-year, 3-year, and 5-year DCA decision curves for the best model RSF at the same period.

**Table 2:** Two types of laryngeal cancer are performed using various survival prediction algorithms.

Model	C-index	IBS			AUC		
		1-Year	3-Year	5-Year	1-Year	3-Year	5-Year
Glottic laryngeal carcinoma							
CoxPH	0.650	0.078	0.171	0.202	0.750	0.708	0.706
RSF	**0.687**	0.116	0.182	0.195	0.730	0.712	0.703
XGBoost	0.651	0.082	0.187	0.229	0.660	0.660	0.681
GBM	0.655	0.083	0.219	0.241	0.719	0.719	0.712
Deepsurv	0.676	0.076	0.168	0.206	0.709	0.709	0.727
Non-glottic laryngeal carcinoma							
CoxPH	0.638	0.150	0.230	0.227	0.656	0.644	0.650
RSF	**0.657**	0.130	0.215	0.220	0.783	0.700	0.682
XGBoost	0.644	0.147	0.229	0.235	0.696	0.682	0.693
GBM	0.647	0.149	0.241	0.244	0.692	0.670	0.674
Deepsurv	0.633	0.160	0.225	0.215	0.688	0.669	0.684

Significant values are in bold.

### Visualization of the optimal model's evaluation indexes

**Figure 6:**
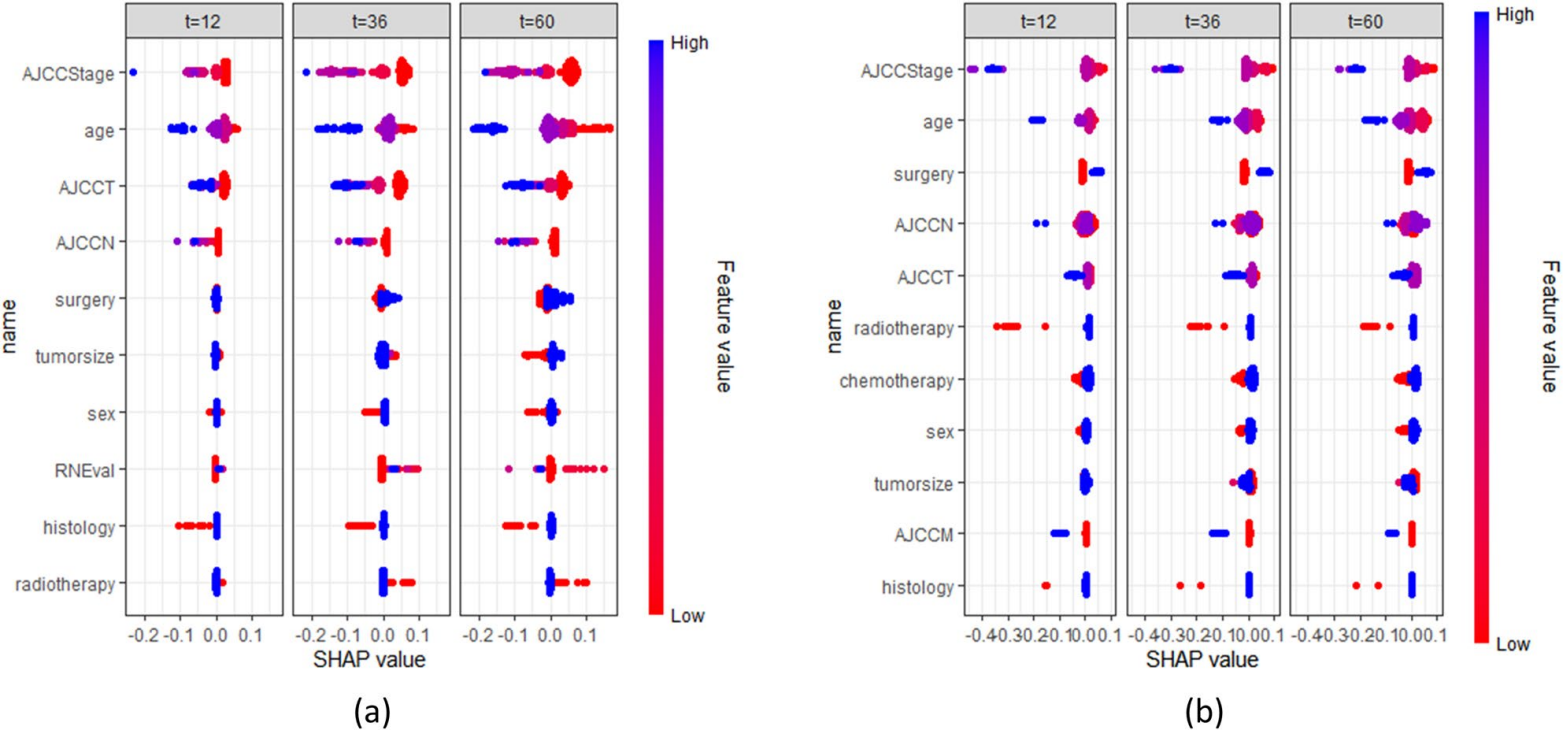
The SHAP plot of the RSF model. The vertical axis lists many clinical characteristics, while the horizontal axis shows how the variable affected the outcomes. The likelihood of dying increases with a feature's SHAP value. The picture reflects the Shap values predicted by 1-year,3-year, and 5 years. (**a**) glottic carcinoma, (**b**) non-glottic carcinoma.

The RSF model is the most effective one for both glottic and non-glottic carcinomas, and its C-index in the test set is 0.687 for glottic and 0.657 for non-glottic, respectively. Their 1-year, 3-year, and 5-year IBS were 0.116, 0.182, and 0.195 for glottic carcinomas, and 0.130, 0.215, and 0.220 for non-glottic carcinomas, respectively. Figure 6 depicts the impact of several clinicopathological characteristics on patient survival for the RSF model of two subtypes of laryngeal cancer. AJCC Stage, age, and AJCC T are the first three factors that have the greatest impact on glottic carcinoma. And it is AJCC Stage, age, surgery for non-glottic cancer.

### The particular forecast

Two patients with glottic carcinoma and two individuals with non-glottic carcinoma were chosen at random. Their clinicopathological data is listed below.

Glottic cancer: Patient 1 is a male, aged 70 to 79 years, with non-squamous cell carcinoma, T3N0, AJCC III, undergoing surgery and radiotherapy; tumor size is unknown; Patient 2 is a male, aged 60 to 69 years, with squamous cell carcinoma, T3N2c, AJCC IVA, not undergoing surgery and undergoing radiotherapy; tumor size is less than 1 cm.

**Figure 7:**
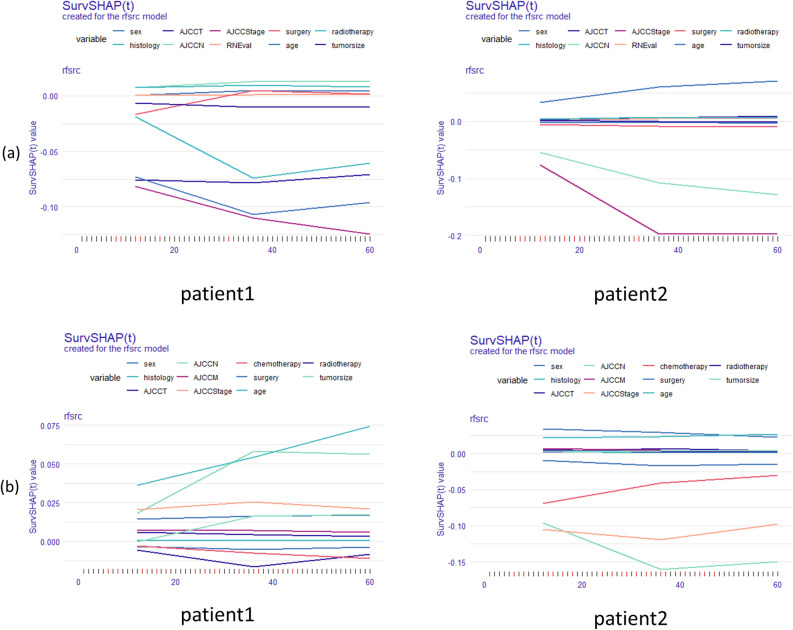
Survival prediction of individual patients. The horizontal axis indicates various ages (1-year, 3-year, 5-year), the vertical axis shows the contribution to survival, while the lines of various hues represent various clinical parameters. Less than 0 indicates a detrimental contribution to survival, whereas more than 0 indicates a beneficial one. (**a**) glottic carcinoma, (**b**) non-glottic carcinoma.

Non-glottic laryngeal carcinoma: Patient 1 is a male, 50–59 years old, squamous cell cancer, T1N0M0, AJCC I, surgery, radiation, chemotherapy, tumor size less than 1 cm; Patient 2: Male, 50–59 years of age, squamous cell carcinoma, T2N3M0, AJCCIVB, surgery, radiation, no chemotherapy, tumor size less than 1 cm. Figure 7 depicts their unique forecasting chart.

## Discussion

In otorhinolaryngology, head and neck surgery, laryngeal carcinoma is a common malignant tumor. Early laryngeal cancer has an occult quality. As examination and treatment techniques advance, the fibrolaryngoscope, for instance, is being used more frequently in clinics to play a significant role in early screening for laryngeal cancer. Nevertheless, since many patients ignore early symptoms such as hoarseness and throat discomfort, many people will mistake them for chronic diseases including chronic pharyngitis, leading to delayed diagnosis and treatment. More than 60% of patients are diagnosed with advanced cancer, based on studies, which significantly lowers the efficiency of laryngeal cancer treatment^2^.

Despite postoperative adjuvant radiotherapy and chemotherapy do not have a favorable prognosis, patients with advanced laryngeal cancer with lymph node metastases sometimes undergo partial laryngectomy or even total laryngectomy. Patients with early laryngeal cancer have a decent prognosis, and their quality of life will significantly diminish as a consequence of total laryngectomy^29^. The physical foundation and developmental base of the larynx are unique. Glottic type, supraglottic type, and subglottic type are three subtypes of laryngeal cancer. The glottic area and subglottic region's structure is derived from the storage trachea germ base, whereas the supraglottic region's structure is derived from the oropharynx germ. As a result, these two regions have different fibrous fascia and lymphatic drainage systems. Clinically, glottic and non-glottic cancer have significantly different risks of lymph nodes and distant metastases. The likelihood of survival varies significantly between various forms of laryngeal cancer, too.

There is some research on the likelihood of surviving laryngeal cancer, however, the majority of them use the outdated Kaplan–Meier estimator survival model (Kaplan and Meier, 1958), which is unable to incorporate the patient's variables. The obvious drawback of the KM survival model is that diverse clinicopathological variables influence how the tumor develops and evolves^5,30^.

The CoxPH model, which can handle censored and censored data as well as continuous and sub-type variables, is suggested as a solution to the problem of covariable fitting. The most used model for predicting survival, the CoxPH model, measures the effect of covariables on survival time using a partial regression coefficient and risk ratio. CoxPH model's assumptions that the risk rate is constant and that the logarithm of the risk rate is a linear function of the covariable are limiting. If the hypothesis is incorrect, the prediction will be biased^8,31^.

Machine learning-based survival analysis has been increasingly used in recent years to forecast the survival of tumor patients. Machine learning can handle complex, nonlinear data, extract relevant features and information, and enhance the model's generalizability and accuracy. Machine learning does not need to make as many assumptions about data distribution or risk functions as the CoxPH model requires. More significantly, we can estimate each patient's survival after the development of a machine-learning model, which has enormous clinical importance. Different machine learning algorithms can be used to handle various types of data and have varying properties. Based on the CoxPH model's survival analysis, RSF, XGBoost, GBM model, and deep learning model Deepsurv are added in this work.

Many academics have discovered that RSF is a survival analysis model with good performance in earlier investigations. It creates several decision trees by self-sampling and combines the outcomes of each tree's predictions by voting or averaging^32,33^.

Of course, similar machine learning studies on the survival analysis of head and neck malignancies, such as laryngeal cancer, hypopharyngeal carcinoma, oropharyngeal carcinoma, and nasopharyngeal carcinoma, have been conducted by various researchers^34,35^. However, as previously mentioned, different embryonic sources and fibrous fascia tissues cause laryngeal carcinoma to be divided into different subtypes. Because previous studies have not distinguished between different subtypes, there will inevitably be a discrepancy between the expected results and the actual results. Based on this, we created the five survival analysis models mentioned above, one for glottic carcinoma and the other for non-glottic carcinoma. RSF is an excellent model, to sum up. The C-index of two separate subtypes of laryngeal carcinoma RSF reached 0.687 and 0.657, respectively, in the final test set. The integrated Brier score (IBS) of their 1-year, 3-year, and 5-year time points is, respectively, 0.116, 0.182, 0.195 (glottic type), and 0.130, 0.215, 0.220 (non-glottic type). This demonstrates the RSF model's high degree of reliability and strengthens our conclusion. The SHAP plot can also more easily convey how risk factors affect specific survival outcomes when compared to the conventional CoxPH analysis nomogram plot. Furthermore, using the RSF machine learning model, we can build the individual survival probability curve for any patient and display their survival prognosis in a more precise manner. This raises the study's clinical relevance even further.

This study has some limitations. First of all, glottic carcinoma and supraglottic carcinoma account for the vast majority of subtypes of laryngeal carcinoma, and because there is a dearth of data on subglottic type, we are unable to develop a survival analysis model for subglottic carcinoma alone. It can only be split into glottic type and non-glottic type as a result. Theoretically, a more precise division results in a more precise forecast. Second, while not terrible, our model C-index still has to be refined by academics.

In conclusion, we compared the prognostic value of patients with various subtypes of laryngeal cancer using five survival prediction model algorithms, and we selected the best RSF algorithm based on which we established survival prognosis prediction for patients with two subtypes of laryngeal cancer, model it and depict it. To advance customized medicine, we also give professionals a tailored patient prognosis prediction model at the same time. Our research demonstrates that the RSF algorithm offers promising therapeutic potential for the prognostic prediction of laryngeal cancer.

### Supplementary Information

Supplementary Table S1.

Supplementary Table S2.

## Data Availability

The data used in this study are available from the Surveillance, Epidemiology, and End Results Program (SEER) database (Incidence-Seer Research Plus Data 17 Registries Nov 2021 Sub).

## Abbreviations

CoxPH: Cox proportional hazards
RSF: Random survival forest
GBM: Gradient boosting machine
XGBoost: EXtreme gradient boosting
MLTs: Machine learning techniques
SEER: The surveillance, epidemiology, and end results program
IBS: Integrated brier score
LASSO: Least absolute shrinkage and selection operator
C-index: The concordance index
